# Medical Opinion and Movement

**Published:** 1908-07-04

**Authors:** 


### Medical Opinion and Movement.

SIR WILLIAM WHITLA discusses at great length and with admirable lucidity in his Cavendish lecture to the West London Medico-Chirurgical Society the complicated question of the distinction between the organisms of bovine and human tuberculosis. After explaining the experiments on which Koch and Schutz founded the former's doctrine of a fundamental difference between the two organisms and of the impossibility of transferring the disease from man to bovines, he dealt in detail with the important researches of Kossel, Weber, and Heuss, which have been held to support those contentions. Several serious objections to such an inference were propounded, and it was shown that in any case Koch's disciples have had to abandon some parts of his position. Beyond that, Sir William quoted the work of many investigators whose results do not support those of the German Commission. Broadly he contends that there are two types of tubercle bacillus, with slightly different cultural, morphological, and pathogenic properties; but he does not admit that these differences constitute them separate species, nor that either type may not by suitable means become modified into the other. These two, which alone for all practical purposes infect the mammalia with tuberculosis, are the human and the bovine types. The latter is found in from ten to twenty-three per cent. of tuberculous lesions in human beings, chiefly in gland and abdominal disease and in children. This type when inoculated into oxen produces results identical with those caused by inoculating bacilli of the same character got from bovine lesions. The other, or human type, is said not to produce these same results, but Sir W. Whitla contends that in reality it does so: the discrepancy among the observations hangs entirely on the matter of dosage, many more human than bovine bacilli being required to set up generalised tuberculosis in the ox.

HAVING got thus far, that human and bovine tuberculosis and their micro-organisms are practically identical, he next considers the mode of infection; this question he narrows down to two probable routes, the respiratory tract, and the intestinal surface. The pith of the lecture is contained in this sentence, "The intestinal route plays a far more important rôle in the production of human tuberculosis than has been hitherto recognised." This important proposition is in direct antagonism to the emphatic pronouncements of Cornet and Koch, who uphold the inhalation hypothesis for the great majority of cases. The experiments on which the latter view is based are carefully examined and criticised in the light of the recent researches of Calmette, Guerin, Vansteenberghe, and Grysez, confirmed and extended by Sir William and Professor Symmers. It has been thus demonstrated that anthracosis of the lungs can be experimentally produced in animals which have never breathed an atmosphere of carbon particles, but have merely had such particles introduced into the intestine; and it is similarly established that tubercle bacilli can and do reach the lungs by the same route in animals without being arrested by the intestinal glands. The series of experiments on which this conclusion is based is long and intricate, but it is very striking and convincing. In Sir W. Whitla's opinion the contention of Calmette that in the immense majority of cases pulmonary tuberculosis is not contracted by inhalation will be generally accepted at no very distant date. The importance of this doctrine and its bearings upon the conduct of the crusade against tuberculosis are obvious and far-reaching, and the Cavendish lecturer deserves the thanks of the profession not less than of the West London Medico-Chirurgical Society for his masterly presentation of the evidence and arguments which constitute his case.

---

IN an important memoir (*Beitrage zur Klinischen Chirurgie*), Reich gives a complete study of accidental and operative traumata of the vagus nerve in the neck. The principal conclusions of his work are as follows: (1) A distinction must be made between simple section of the nerve (which merely causes paralysis), and other injuries such as crushings and contusions, which excite it. (2) The operation of simple unilateral vagotomy is without danger, and causes no immediate or remote serious symptoms, except paralysis of the corresponding vocal cord. Slight symptoms, which have no effect on the ultimate prognosis, may be noticed, such as tachycardia, and slowing and increased forcefulness of respiration. (3) Certain post-operative complications, occasionally noticed, are explicable as being due to other lesions, as well as to the gravity of the operation. Such are dysphagia, bronchitis, pneumonia, etc. (4) Death does not follow simple vagotomy, but the ends of a divided nerve should nevertheless be sutured together. (5) Crushing of the nerve, however, is followed by alarming symptoms, momentary inhibition of cardiac and respiratory action, which may end in death. These symptoms of excitation are the result of reflex action upon the cardiac and respiratory centres in the bulb. (6) To combat them the nerve may be cocainised above and below the lesion; if this fails to relieve symptoms it must be divided. These conclusions are supported by the author's experiments on animals, with the reserve that man is more susceptible to excitation of the nerve than animals, for it was found impossible to cause an animal's death by simple excitation, however great the stimulus.

---

PETIT and Minet, in the *Société de Biologie*, have published the results of their inquiries into the power of the rectum to absorb egg-albumen. At intervals of a week a solution containing half the white of an egg was injected into the large bowel of several rabbits. By the end of a month the animals were rapidly losing flesh, so they were bled and their serum collected. This, diluted to 1 in 10,000, was found to precipitate a solution of 1 in 10 of egg-albumen. Serum obtained from control-rabbits showed no such tendency. Next the white of an egg, diluted with 50 c.c. of water, was injected into the rectum of an organically sound man. The patient's urine was collected and examined hourly. On treating this urine with the serum obtained from the rabbits a precipitate was formed, a phenomenon which commenced to show itself at the end of the first hour, only disappearing at the end of 24 hours. It was impossible by the ordinary means of boiling, nitric acid, etc., to demonstrate the presence of albumen in the urine. Serum obtained from the patient and mixed *in vitro* with that obtained from the rabbits gave no such precipitate when added to the patient's urine. The observers conclude, as the result of these experiments, that absorption of albumen takes place naturally by the rectal route. The interest of these observations lies in the fact that of late the utility of rectal-feeding has been much disputed. If the conclusions arrived at be correct, the practice of rectal-feeding, at any rate with solutions of egg-albumen, would seem to be justified.

---

As the result of recent observation, it is known that a spontaneous evacuation of gas from the stomach does much to relieve the painful crises observed in persons suffering from certain heart affections, and this clinical fact is not surprising, seeing that the same nerve supplies both viscera, and that reflex cardiac troubles are common in those suffering from gastric disturbances. Herz of Vienna has, by means of the following procedure, attempted to relieve the agonising crises of angina by causing the patient to eructate. The patient, in a sitting position, is made to drink, and keep in his mouth, a small quantity of water (a large quantity is useless, as it would merely tend to dilate the stomach), then to throw his head as far back as possible, at the same time swallowing the mouthful of water. In this position, the œsophagus being well stretched, a certain sensation is produced which, according to Herz, is the cause of the eructation which follows the end of the act of deglutition. It is as well to warn the patient of the result desired, so that he may not for any reason prevent the eructation, but rather try to produce it. By this simple manœuvre Herz claims to have arrested a number of anginal attacks, and says that if the procedure be followed as soon as prodromal symptoms make their appearance, an attack may be aborted altogether. In several cases of paroxysmal tachycardia the attacks have been sensibly diminished in violence, and the pulse slowed, immediately after the stomach has, by this procedure, been emptied of gas.

---

After a systematic analysis of the urine of several hundred patients suffering from psoriasis, Bulkley (Journ. of Amer. Med. Assoc.) has come to the conclusion that the affection is accompanied by profound disturbance of the various organic interchanges. Thus the reaction of such urines is from twice to four times as acid as that of normal urine; their density varies from 1030 to 1040, and the urea content is about double the normal. Meat diet, which accentuates the disorders of nutrition, aggravates the eruption. On the other hand, a strictly vegetarian diet (from which eggs, milk, and fish are rigidly excluded) has an entirely different effect. According to the author's experience, such a change of diet causes the eruption to fade and become less squamous, and in a few weeks to disappear entirely, without any local treatment whatever. It is scarcely necessary to mention that the treatment must be carried out for a considerable length of time, or the eruption will tend to reappear. This necessity for lengthy treatment does not, however, seem to weigh heavily upon the patient, for as a rule he soon becomes used to the change, and Bulkley knows several patients who, once their psoriasis had been cured, still remained faithful to their new diet. The treatment is simple and inexpensive, but somewhat monotonous.

---

THE close associations between rheumatic fever and chorea on the one hand and rheumatic fever and an infective condition of the throat on the other have been recognised for a long time. M. de Ponthière maintains, as a result of careful observations carried out for some years, that chorea is also closely associated with unhealthy and enlarged tonsils and adenoids. He is of opinion that chorea is usually a symptom of an auto-intoxication produced by the absorption of septic material formed in the naso-pharynx, the infection being usually of a rheumatic nature. He finds that removal of the tonsils and adenoids in such cases is invariably followed by cessation of the choreic symptoms. He points out the analogy between the symptoms of the adenoid condition and those of the choreic patient; defective respiration, disturbed nights, night screams, dyspnœa, defective intelligence, heavy aspect, digestive troubles, etc. The comparison is probably somewhat overdrawn and exaggerated, but it is fairly well established that the rheumatic infection takes place in a large proportion of cases through the throat, and an unhealthy condition of the naso-pharynx may probably keep up the infection as well as offer a source of reflex irritation by which the peculiar choreic manifestations may be excited.

---

M. LANCEREAUX has for many years held the view that arterio-sclerosis is a manifestation of gout, and is dependent upon a deficient functional condition of the sympathetic nerves, by which the arterial tissues are affected in a secondary manner. At a recent meeting of the Academy of Medicine he has again brought these views forward. He denies altogether the usual causes to which this disease is attributed, such as old age, alcohol, tobacco, or syphilis. He points out that arterio-sclerosis never commences after about sixty-five, and that to many afflicted with the disease the usually assigned causes cannot be attributed. In support of his theory he cites the experiment of Giovanni, in which the sympathetic was cut close to the vertebral column, and subsequently sclerotic plaques appeared in the aorta. M. Chantmesse's observation is also quoted in support of this theory; a local arterio-sclerosis limited to the arm followed an injury to the shoulder, causing damage to the brachial plexus. M. Lancereaux regards arterial high tension as the result of the disorder, and not the cause, and deprecates any treatment based on its reduction. He advocates the regular administration of iodide of potassium and thyro-iodine, and lays little stress on any rigid restrictions in diet.

RAPID advances in knowledge by experimental research on new lines are apt to dazzle by the new light they throw on the field, and so obscure and hide from our view the vast area of ground as yet unexplored and undiscovered. It is very necessary, therefore, that we should be reminded from time to time of the great gaps in our knowledge, in order that we may be stimulated to further efforts to fill these gaps. Professor Sir W. Watson Cheyne has performed this task in a highly interesting and suggestive address on the defensive arrangements of the body as illustrated by the incidence of disease in children and adults. It is a matter of common knowledge that marked variations occur in the incidence of diseases at different ages. Some diseases, in particular the exanthemata, occur chiefly during childhood, while other diseases which are common in adult life hardly ever affect children. And again, certain diseases which attack all ages are more virulent at one age than at another. The explanation of these facts probably lies in differences in the resisting power of the body to the invading organism, but this only carries us one step further towards the goal. We still have to seek an answer to the question, in what these differences of resisting power consist? In endeavouring to answer this question Sir Watson Cheyne lays stress on the local resisting power of the individual tissues rather than on the general bactericidal properties of the blood. The first line of defence of the body resides probably in the epithelial tissues, and many instances are cited in which there appears to be a varying degree of resisting power to specific lesions at different ages. Pyogenic organisms behave differently in the skin of children and adults. In children multiple abscesses of the skin and impetigo are common, but boils rarely occur, while the reverse holds for adults. Such differences may possibly depend upon anatomical variations either in the epidermis itself or in the underlying tissues.

FOLLOWING the invasion of the micro-organisms still further, one may suppose their arrival into the blood-stream, whence they either find a resting-place in the body or are overcome and die out. The endothelial lining of the blood-vessels Sir Watson Cheyne regards as the second line of defence. It is here that in many diseases the specific lesion has been shown to take its origin, for example, in tuberculosis and syphilis. The question now arises, What determines the invasion by the microbe of some particular part or tissue of the body? Why does the gonococcus select the urethra and the conjunctiva? Why does the tubercle bacillus show a predilection for the lung tissue, the glands, and in children bones and joints? A plausible suggestion is that these several tissues offer a pabulum specially suitable to the specific microbe. What the characteristics of these pabula are remain at present unknown. But these and many other equally cogent points emphasise the importance of the local conditions of the tissues and their variations both at the same age and at different ages as determining factors in the invasion of the body by the bacteria of disease. Many will sympathise with Sir Watson Cheyne in the opinion that while the extensive work on opsonins is important and interesting, it would be more convincing if the theories were less complete and simple and the writings tinged with a little philosophic doubt. He is convinced there is still something wanting, and suggests that the discovery of that something may be made by studying rather these local conditions of resisting power than the general conditions of immunity.

STAEHELIN, of Basel, has collected the various cases of primary gastric sarcoma, and comes to the conclusion that the clinical differentiation of gastric sarcoma from gastric carcinoma is of little importance and only of theoretical interest. In a case he reports the diagnosis was "carcinoma ventriculi," but at the autopsy a sarcomatous tumour was found, which, on microscopical examination, was declared to be a primary round-celled sarcoma. He has collected twenty-two somewhat similar cases from the literature, and added ten cases of primary spindle-celled, twenty-three of lympho-, twelve of myo-, and ten of fibro- and myxo- sarcomata. The clinical history is usually a very long one, and the examination of the stomach contents, as in the case of carcinoma, reveals an absence of free hydrochloric acid and the presence of lactic acid. Death is usually due to inanition, and peritonitis from direct perforation is not common. The treatment is purely operative, and the prognosis, in cases in which the growth is localised and not of the diffuse variety and which are operated upon at an early stage, is relatively good. In twenty-four recorded cases of fairly-wide extirpation, in eleven no recurrence of the growth has been observed.

LEOTTA reports, in the surgical section of *Il Policlinico*, his researches on the ligature and suture of large veins. His paper brings forward nothing that is essentially new, but draws attention once more to the fact that surgical interference, in the case of injuries to the large venous trunks, is less dangerous and more promising than older surgeons believed. Thus he found that ligature of the superior vena cava, above the azygos major veins, is not followed by untoward after-effects in about 75 per cent. of cases, the circulation being carried on by means of the azygos, the superior intercostal, and the iliac and epigastric veins communicating with the internal mammary vein. Ligature of the inferior vena cava above the renal veins is invariably fatal (by suppressing the renal function) if done suddenly. For the same reason ligature of both renal veins is fatal in 30 per cent. of cases; in the others the anastomosis between the ascending lumbar, the reno-azygos vein of Legars and the internal mammary veins suffices after a time to carry on the circulation. Ligature of the inferior cava below the influx of the renals is usually well born and gives rise to no serious after-effects. In all these cases the local venous stagnation was marked.

